# Design of intelligent controller for obstacle avoidance and navigation of electric patrol mobile robot based on PLC

**DOI:** 10.1038/s41598-024-63810-5

**Published:** 2024-06-12

**Authors:** Zhenfang Liu, Mengyuan Li, Dongshuai Fu, Shuai Zhang

**Affiliations:** Hebei University of Water Resources and Electric Engineering, Cangzhou City, 061001 Hebei Province China

**Keywords:** Laser range finder, Electrical patrol inspection, Mobile robot, Obstacle avoidance navigation, PLC control, Controller, Computational science, Engineering

## Abstract

Currently, the obstacle avoidance control of patrol robots based on intelligent vision lacks professional controller module assistance. Therefore, this paper proposes a design method of intelligent controller for obstacle avoidance and navigation of electrical inspection mobile robot based on PLC control. The controller designs a laser range finder to determine the required position of electrical patrol inspection. Use PLC as the core controller, and combine sensors, actuators, communication module and PLC selection module in the process of hardware design to achieve autonomous navigation and obstacle avoidance functions of the robot. Then design the software including the PLC compiler system and the virtual machine module. Based on the above steps, design the control module of obstacle avoidance navigation, which realizes the key link of robot autonomous navigation. The test results show that the controller can successfully avoid obstacles, improve the efficiency and quality of inspection, and achieve accurate and fast obstacle avoidance navigation for the electrical inspection mobile robot.

## Introduction

Electrical inspection mobile robots are robots that can be automated or remotely controlled to perform inspection and maintenance work in power systems. They are usually equipped with a variety of sensors and devices to detect the status and operation of electrical equipment and perform the necessary maintenance and repair work^1^. Electrical inspection mobile robots can be adapted to different environments and tasks, and can carry out inspections in complex and hazardous environments, reducing human intervention and risk. They can also receive and send data through wireless communication technology^2,3^, and transmit inspection results and fault information to control centers or cloud platforms, helping staff to better understand the status of equipment and maintenance work. The application of electrical inspection mobile robots can greatly improve the reliability and safety of the power system^4^, reduce the error and risk of manual inspection, and improve work efficiency and maintenance quality. At the same time, they can also help the staff to better understand the state and operation of the equipment, providing important data support for the optimization and improvement of the power system.

In the first section, the paper introduces that traditional obstacle avoidance and navigation methods have problems in robustness. Their performance is vulnerable to changes in different environments and tasks. Therefore, aiming at the above problems, a design method of intelligent controller for obstacle avoidance and navigation of electrical patrol mobile robot based on Programmable Logic Controller (PLC) is proposed to improve the adaptability and reliability of mobile robot. In the second section, provide a literature review on relevant solutions. In the third section, design the robot position laser ranging module to obtain the position information of the robot relative to the environment in real time. Then design the overall program including the design of hardware and software. During the process, use PLC as the core controller, and combine sensors, actuators, communication modules and other components. Make virtual machine and PLC compiler work closely together to realize data transmission and feedback through communication interface, and at the same time provide a visual interface, so that developers can intuitively understand the running state and control effect of the robot. In the forth section, the design method shows good adaptability and robustness in complex real-world environments, can successfully avoid obstacles, and improves the safety and accuracy of robot operation. From the experience, it shows that the speed and acceleration and other aspects of the method have obvious advantages, and it can also achieve accurate and rapid obstacle avoidance navigation in a variety of situations.

## Review of references

Obstacle avoidance navigation technology of electrical inspection mobile robot is one of the key technologies to realize its autonomous movement. Relevant scholars have carried out a lot of research on this, such as Reference^5^ proposed a robot obstacle avoidance method based on deep reinforcement learning, and trained a model to learn direct control commands from the original depth image through self-monitoring reinforcement learning algorithm. The virtual reality platform is used to simulate the natural working environment and generate obstacle avoidance data for training. Through the obstacle avoidance simulation of virtual robots in virtual environments, the automatic domain randomization technology is used to generate randomly distributed environmental parameters, which helps to obtain better performance in natural environments. The results of this paper show that the proposed method can effectively make the robot have safety awareness and learn how to change its trajectory to avoid accidents with people in the workspace. Reference^6^ proposed a neural network based obstacle avoidance for mobile robots based on standardization technology, and used the navigation method based on artificial neural network to train data sets to provide high mean square error from training on MATLAB Simulink tool. When it comes to knowledge-based systems, artificial neural networks can be well adapted in an appropriate form. Reference^7^ proposed that the experimental evaluation of robot obstacle avoidance using deep reinforcement learning, relying on the equivalent linear parameter change state space representation of the system, and according to the measurement of the distance between the robot and the obstacle, activate two operation modes, one based on joint position and speed, and the other based only on speed input. Therefore, when the obstacle is close to the robot, a switching mechanism is introduced to enable the Deep Reinforcement Learning (DRL) algorithm, thus generating a self configuring architecture to deal with objects moving randomly in the workspace. The hybrid collision avoidance strategy based on DRL is tested on EPSON VT6 robot, and satisfactory results are obtained. Reference^8^ proposed a heuristic algorithm for obstacle avoidance of continuum robots based on FABRIK, which modeled the update of key nodes as the movement of electric charges in the electric field, avoiding complex nonlinear operations. The robustness of inverse kinematics and path tracking in complex environments is achieved by imposing constraints on key nodes and predetermining the position of obstacles. The algorithm is characterized by fast convergence speed, low computational cost and can be used for real-time applications. The proposed method also has wide applicability, and can be applied to continuous robots with mobile and fixed bases. And it can be further extended to the field of super redundant robots. By simulating the path tracking and obstacle avoidance of the five segment continuum robot in different environments, and comparing with the classical methods, the effectiveness of the algorithm is further verified. Reference^9^ proposed a real-time obstacle avoidance algorithm for robots based on the prediction of the reachable area of obstacles, established the dynamic obstacle status update prediction equation, and realized the prediction of the reachable area of the dynamic obstacle. For dynamic and static obstacles, the multi-step elliptical envelope potential field based on the prediction of the reachable area and the potential field based on the new sigmoid function were proposed respectively, and the logarithmic Lyapunov gravitational field of the target was modified, A real-time obstacle avoidance algorithm for robots in multi type obstacle space is presented. Numerical simulation and experimental results show that,Real-time obstacle avoidance algorithms can lead to shorter path lengths, higher safety and smaller amplitude of maximum travel angle variation during obstacle avoidance. Reference^10^ proposes a co-optimization strategy for distributed multi-robot manipulation with obstacle avoidance and internal performance maximization, where a decentralized optimization process is first run to generate a robot position reference, and then a joint level of control is responsible for tracking. The planning phase also includes the necessary internal performance optimization since the available workspace for each robot is more constrained in a multi-arm manipulation system. The velocity-level optimization problem will be solved and then translated into a position-level control reference so that it can be combined with a number of compatible interactive controllers. Simulations and experiments are carried out on two redundant manipulators to validate the proposed design, and the data show that on-line computation is fast.

## Design of obstacle avoidance navigation controller for electric patrol mobile robot based on PLC

### Robot position laser ranging module design

The positional laser ranging module of the power inspection robot has ultra-high resolution and accuracy, and it has incomparable advantages in angular resolution, acquisition efficiency and anti-interference performance. Robot position laser ranging can accurately identify the target point^11,12^ in the robot's surroundings, and collect the distance, direction and other information, which is expressed in the form of polar coordinates as follows.

1 $$ W_{D} = \left( {X_{m} ,Y_{m} } \right) $$

In Eq. (1),$X_{m}$, $Y_{m}$ denote the position point of the target point in the direction of horizontal and vertical coordinates, respectively.$m$ indicate the number of target points. Express the result in the form of a Cartesian coordinate system as.

2 $$ W_{D}^{\prime } = \left( {W_{X} ,W_{Y} } \right) $$

In Eq. (2),$W_{X}$, $W_{Y}$ denote the horizontal and vertical coordinates of the laser ranging position, respectively.

Assuming that the line segment feature of the original laser scan data^13,14^ is expressed in polar coordinate system as $\left( {\theta ,\lambda } \right)$, then the expression in the right-angled coordinate system is:

3 $$ \cos \lambda + \sin \lambda - \theta = 0 $$

**Figure 1:**
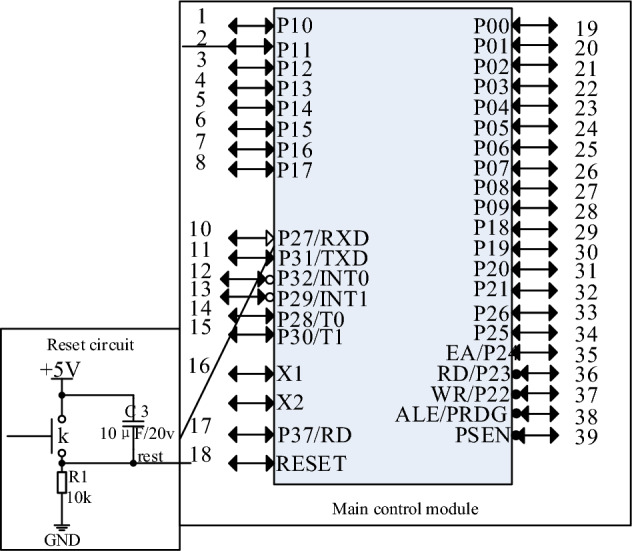
Circuit diagram.

In the design of the robot position laser ranging module, the circuit diagram is one of the key aspects, describing the connections between the components within the module and the electrical signal transmission paths, the circuit diagram is shown in Fig. 1.

Figure 1 describes the connection mode and electrical signal transmission path between each component. On this basis, considering the interference^15^ generated by the position laser ranging controller of the power inspection robot, assuming that d_*n*_ is subject to the Gaussian white noise distribution, the distribution of laser data noise can be obtained by using the error transfer formula:

4 $$ P_{n}^{{}} = \varphi_{n} \times \sigma_{d}^{{}} \times \cos \lambda + \sin \lambda $$

In Eq. (4),$\varphi_{n}$ denotes the error coefficient, the $\sigma_{d}^{{}}$ denotes the distance value under Gaussian white noise distribution. The weighted total length of all co-located points is calculated by Eq. (5):

5 $$ L\left( \theta \right) = \frac{{P_{n} \times \left( {W_{D} + W_{D}^{\prime } } \right)}}{{L_{a} + L_{b} }} $$

In Eq. (5),$L_{a}$ indicates the start point of the laser line segment.$L_{b}$ denotes the termination point that does not satisfy the characteristic straight line criterion. The design of the robot position laser ranging module is based on one of the key components of robot navigation and localization, which obtains the position information of the robot relative to the environment in real time through laser ranging technology. Through the reasonable design, it can realize the precise positioning and navigation of the robot in the complex environment, improve the motion control and path planning ability of the robot, so as to realize the efficient, stable and safe robot operation.

### Design of obstacle avoidance navigation control module based on PLC

#### Overall design program

The obstacle avoidance navigation control module designed in this paper uses PLC as the core controller, and combines sensors, actuators, communication modules and other components to achieve autonomous navigation and obstacle avoidance functions of the robot. The specific design scheme is as follows:

1. Sensors: Infrared ranging sensor^16^ , ultrasonic sensors^17,18^ , etc. are used to sense the environment information around the robot in real time, such as the distance to the obstacles and the traveling path.
2. Actuator part: including motor driver, steering gear, etc. According to the control signal of PLC, drive the robot to travel according to the planned path.
3. Communication module: RS485, Ethernet and other communication modes are used to realize data transmission and command control between PLC and upper computer and other equipment.
4. PLC control part: according to the environmental information collected by the sensor, through algorithm processing, output control signals to the actuator to achieve autonomous navigation and obstacle avoidance of the robot.
5. Application program: including environment perception and modeling, navigation control.

#### Hardware design

The software design of PLC compiler and virtual machine plays an important role in the research of PLC intelligent control method for obstacle avoidance navigation of electric patrol mobile robot. As a development tool for control programs, PLC compiler supports multiple programming languages, generates machine-executable codes through syntax parsing and compilation, and provides rich functional block libraries to simplify the development process. During the compilation process, the compiler can also diagnose errors to help developers quickly locate and fix problems. The virtual machine is responsible for simulating the running environment of the robot, simulating the motion state and response of the robot in real time, and providing a platform for developers to test and verify programs without actual hardware. Virtual machine and PLC compiler work closely together to realize data transmission and feedback through communication interface, and at the same time provide a visual interface, so that developers can intuitively understand the running state and control effect of the robot.

(1) Sensor selection: according to the actual demand, choose the appropriate infrared ranging sensors and ultrasonic sensors to ensure that the sensors can sense the environmental information stably and accurately. Electrical inspection mobile robot movement as a three-dimensional movement in space, three-dimensional movement abstracted into two-dimensional activities, electrical inspection mobile robot kinematics involved in the description of each parameter^19^ , the expression formula for.

6 $$ \left\{ \begin{gathered} x_{i} = v_{i} \times v_{o} \hfill \\ y_{i} = x_{i} /m \hfill \\ \end{gathered} \right. $$

In Eq. (6),$x_{i}$ denotes the horizontal coordinates of the initial position of the electrical inspection mobile robot; the $y_{i}$ denotes the initial position vertical coordinate of the electrical inspection mobile robot; the $v_{o}$ denotes the speed value of the electrical inspection mobile robot; $m$ denotes the quality of the electrical inspection mobile robot. The obstacle mass detected by the electrical inspection mobile robot in the two-dimensional direction is abstracted as a mass with unit mass, at this time, the real-time state value of the electrical inspection mobile robot is.

7 $$ Z_{i} = \left( {x_{i} ,y_{i} } \right)^{{\text{T}}} $$

In general, due to the existence of the drive constraints of the electrical inspection mobile robot itself, there is also an upper limit value of the movement speed^20^ , which can ensure the effective braking of the robot in the event of an accident. With reference to this situation, we calculate the upper limit value of the running speed of the electrical inspection mobile robot and the average value of the speed of the formation formation, the mean value $\overline{v}_{i}$ between the two is obtained as the reference value of the automatic control to improve the accuracy of the control. The calculation formula is as follows:

8 $$ \overline{v}_{i} = v_{\max } \times S\left( t \right) \times Z_{i} $$

In Eq. (8),$S\left( t \right)$ indicates the time parameter.$v_{\max }$ indicates the maximum rate value of the electrical inspection mobile robot.

(2) Actuator selection: in the field of electrical inspection, mobile robot as a new type of inspection, with independent navigation, obstacle avoidance, detection and other functions, greatly improving the inspection efficiency and quality. Actuator is a key component in the robot control system, responsible for the conversion of electrical signals into mechanical movement or operation, the selection of high-performance motor drive and servo, to ensure that the electrical inspection mobile robot can be in accordance with the planned path of fast and accurate traveling.

**Figure 2:**
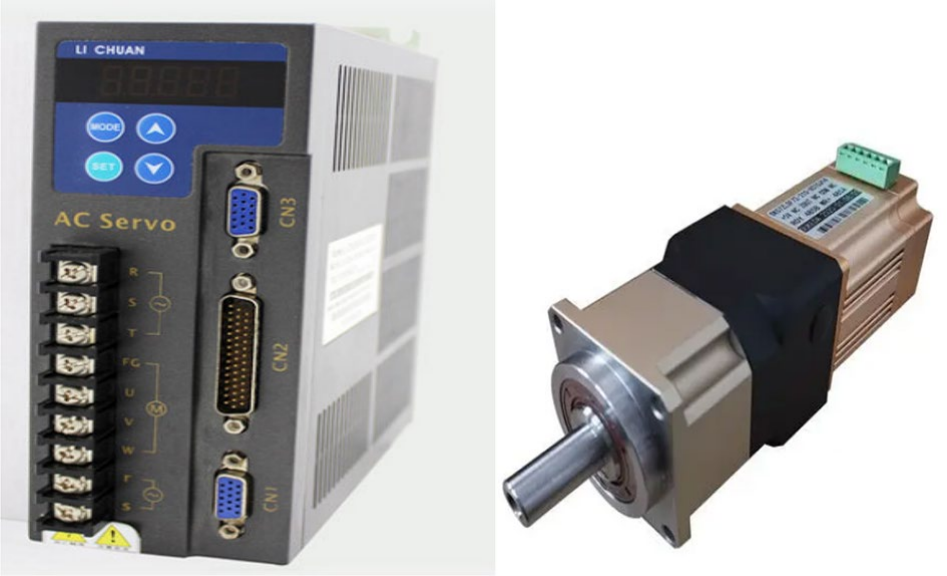
Structure of the motor driver.

Where the motor driver is shown in Fig. 2.

**Figure 3:**
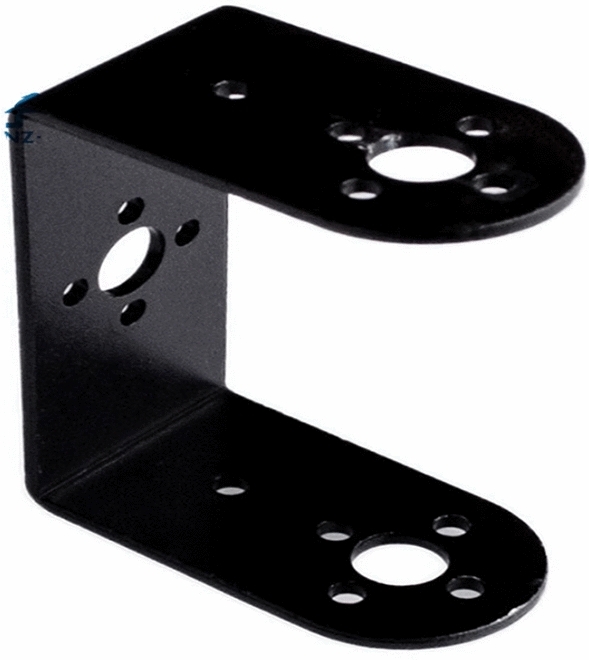
Rudder structure.

The structure of the servo is shown in Fig. 3.

**Figure 4:**
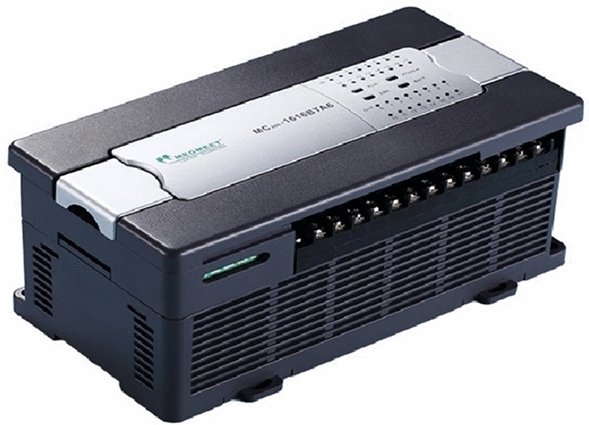
Sensor structure diagram.

The sensor structure with PLC is shown in Fig. 4.

Selecting high-performance motor actuators and servos is an important step to ensure that the electrical inspection mobile robot can travel quickly and accurately according to the planned path. The right actuator can provide strong power and control capability, providing solid support for the robot's motion control and path planning, thus improving the overall performance and reliability of the robot system.

(3) Communication module design: The communication module is an important part of the electrical patrol mobile robot, which is responsible for data interaction and control command transmission with the host computer or other devices. In order to ensure the reliability and real-time of data transmission, stable RS485 communication interface and Ethernet interface are used in the design.

First of all, the RS485 communication interface is chosen because of its strong anti-interference ability and long-distance transmission advantages. With the RS485 interface, the robot can stably communicate with other external devices in serial mode, and transmit and receive data. RS485 supports multi-point communication, and can connect multiple devices, so that the robot can interact with multiple external devices during the patrol process to achieve more flexible control and monitoring.

Secondly, the Ethernet interface plays an important role in the design of the communication module. Through the Ethernet interface, the robot can communicate with the host computer or other network devices in the LAN. Ethernet communication is characterized by large bandwidth, high speed and high stability, which can meet the inspection tasks with high real-time requirements. At the same time, with the help of Ethernet interface, the robot can also be remotely monitored and controlled through the network, which improves the working efficiency and convenience.

In practical application, through the selection of stable RS485 and Ethernet interfaces, the communication module design can ensure the reliable communication between the electrical patrol mobile robot and external equipment, and realize the timely transmission of data and the accurate execution of control commands. This provides a strong support for the robot's patrol task, and improves the overall performance and reliability of the robot system.

(4) PLC selection: select high-performance and reliable PLC to ensure the stability and real-time performance of the control system. At the same time, according to the actual needs, select the appropriate PLC expansion module to meet the system expansion needs.

In the process of obstacle avoidance navigation, the dynamic characteristics of the electric patrol mobile robot are one of the key factors in controller design. When considering the dynamics of mobile robot, the controller based on PLC needs to fully understand the robot's motion characteristics, constraints and the influence of external environment to ensure that the robot can perform the inspection task stably and efficiently.

On the one hand, PLC controller needs to consider the dynamic characteristics of the robot. Including the inertia, friction and gravity of the robot. When planning the trajectory of the robot, the PLC controller needs to fully consider these dynamic factors to avoid the unstable motion of the robot due to excessive acceleration or speed change.

On the other hand, obstacle avoidance navigation is one of the important functions of mobile robots. The PLC controller needs to obtain the environmental information around the robot in real time, and plan the motion path of the robot according to this information. In the process of obstacle avoidance, PLC controller also needs to consider the dynamic constraints of the robot to ensure that the robot can avoid obstacles and maintain a stable state of motion.

The PLC specification table of PLC intelligent control method for obstacle avoidance navigation of electric patrol mobile robot is shown in Table 1.

**Table 1:** PLC specification table.

Specification item	Describe
Model	S7-400
Processor	High speed, low power processor
Communication interface	Support Ethernet, RS-485, USB and other communication modes
Storage capacity	Enough to store control programs, data, etc
Expanding ability	Support IO module, communication module and other extensions
Programming software	Provide easy-to-use programming software and support ladder diagram, instruction list and other programming methods
Real-time performance	High real-time, ensuring quick response to external signals
Working environment	Adapt to the industrial environment, with dust-proof, waterproof, anti-electromagnetic interference and other characteristics
Power requirement	Wide voltage range to meet the power supply requirements of different occasions
Security certificate	Comply with relevant international and domestic safety standards

**Figure 5:**
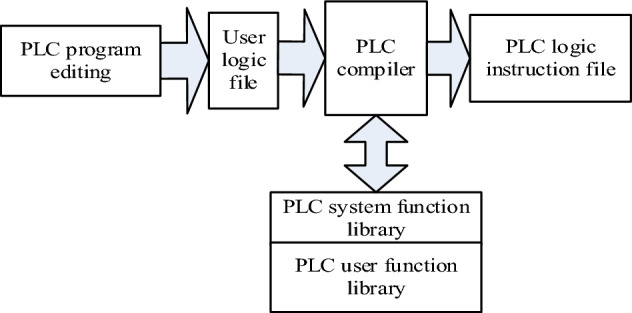
PLC controller structure diagram.

Taking PLC controller as the core hardware, the overall structure of obstacle avoidance navigation control system is designed. The touch screen is used as an operating terminal device to monitor the system operation status, read or write control commands at the same time, and the power supply model is F7PU27-9A to provide support for the normal working mode and low power consumption mode, so that it can still supply power to the PLC system in the power failure state. The data collector configured for the electrical patrol mobile robot is F8LE52-0S, which uses Ethernet interface to communicate with infrared sensors, cameras and other external devices to provide output signals for external devices and conduct solenoid valve control. At the same time, F8NS72-0 V servo motor and F4YC82-0N relay are used to achieve DC input and output. The structure diagram of PLC controller is shown in Fig. 5.

As the core part, CPU processes input signals and executes program logic to control output signals. The input module converts the external signals into digital signals, and the output module converts the digital signals generated by PLC into physical signals and controls the external equipment.

(5) Application program design: Describe the application program part of the development method:

First: environmental perception and modeling.

① Sensor integration: The mobile robot needs to integrate various sensors, such as lidar, ultrasonic sensor, infrared sensor and camera, etc., in order to obtain the information of the surrounding environment.

② Data processing: The data collected by sensors need to be preprocessed and filtered to eliminate noise and improve data accuracy.

③ Environment modeling: Using the obtained data to build a three-dimensional model of the environment, which is helpful for the robot to understand and navigate the environment.

Second, navigation control.

① Positioning and map matching: the robot is accurately positioned by using positioning and map construction technologies, and matched with the pre-built environmental map.

② Path tracking: According to the planned path, the path can be accurately tracked by controlling the motion of the robot.

③ Speed control: adjust the speed of the robot in real time according to the environmental conditions and path requirements, so as to realize smooth and safe navigation.

#### Software design

**Figure 6:**
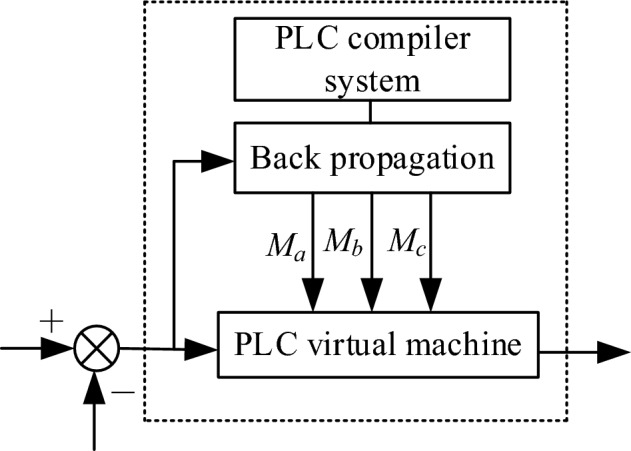
Intelligent controller design schematic.

The system structure block diagram of the intelligent controller for obstacle avoidance and navigation of the electrical patrol mobile robot optimized by PLC technology is shown in Fig. 6, including the PLC compiler system and the PLC virtual machine module.

The specific method of the software part of the intelligent controller for obstacle avoidance navigation of the electrical patrol mobile robot based on PLC is as follows:

Step 1: According to the structure of PLC compiler system and PLC virtual machine, design two input nodes and one output node, and obtain the optimal initial value of weighting coefficient through BP neural network algorithm;

Step 2: Perform dimensionless processing to obtain the input value of the neural network;

Step 3: According to the input and output calculation of each node of the neural network, the three nodes of the output layer are the three parameters $M_{a} ,\,M_{b} ,\,M_{c}$ of the corresponding PID controller;

Step 4: Use the BP neural network algorithm to correct the weighted value of the neural network;

Step 5: Return to Step 2 and repeat to obtain the optimal conversion requirements, and obtain the original weight of the intelligent controller according to the population calculation of PLC compiler system and PLC virtual machine optimization.

To sum up, the software design of the unmanned driving interaction system is completed. Thus, the design of intelligent controller for obstacle avoidance and navigation of electrical inspection mobile robot based on PLC is realized.

## Experimental tests

### Experimental robot and environment

**Figure 7:**
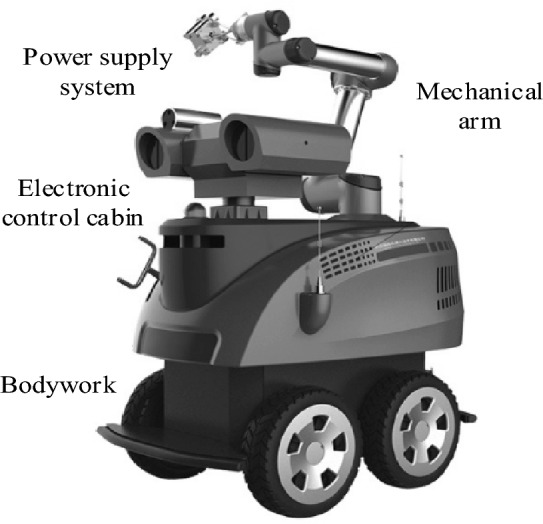
Structural diagram of T7-E wheeled patrol inspection robot.

The intelligent control method of PLC for obstacle avoidance navigation of electric patrol mobile robot is designed, and the performance of the controller is simulated and tested with an electric patrol robot as the experimental object. The model of the experimental electrical patrol mobile robot is T7-E wheeled patrol robot. T7-E wheeled inspection robot is an intelligent inspection robot, and its structure is shown in Fig. 7.

Simulate the real industrial environment in the laboratory with multiple obstacles, use the wheeled mobile robot as the experimental carrier, carry the PLC control system, sensor module and actuator module, use the industrial PLC as the main controller, be responsible for processing the sensor information, and carry out path planning and obstacle avoidance navigation according to the algorithm, and detect the environmental information and obstacle distance around the patrol robot. In order to ensure the effect of PLC intelligent control method for obstacle avoidance navigation of electric patrol mobile robot, the operation parameters of the method are set, including the structure, learning rate, discount fActor, batch size and iteration times of actor network and Critic network. The initial layers of Actor network and Critic network are 25 layers, and the learning rate is an important parameter to control the step size of model weight update, and the initial learning rate is set to 0.001; The discount factor is used to control the importance of future awards, with an initial value of 0.1. Batch size refers to the number of samples used each time the model weight is updated, and iteration times refers to the number of times the model weight is updated in the training process. The initial values of the above parameters are set to 100 and 200 respectively. In the above experimental environment, set the relevant experimental parameters of the patrol robot, as shown in Table 2.

**Table 2:** Experimental parameters related to inspection robot.

Serial number	Parameters	Element
1	Effective inspection time	> 1.5 h
2	Tempo	The maximum moving speed can reach 2 m/s
3	Communication method	Support Wi-Fi and Bluetooth communication, convenient data transmission with upper computer
4	Rated voltage of drive motor	32 V
5	Rated speed	10200r/min
6	Peak torque	1.4N·m
7	Holding torque	0.5N·m

**Figure 8:**
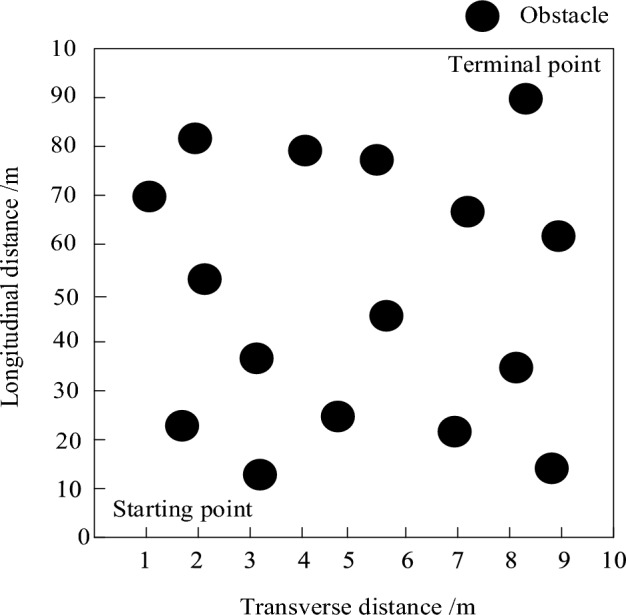
Study area.

According to the working environment and parameter setting of the electrical inspection mobile robot, the 10 m × 10 m area was selected as the research area, as shown in Fig. 8.

According to Fig. 8, the circle in the study area represents the original obstacles in the area. The electrical patrol mobile robot is located at (0 m, 0 m), which is the starting point. After passing through the obstacles in the middle, it finally reaches (10 m, 10 m), which is the end point.

Use VC6.0 to develop the demonstration platform and establish the simulation map of the local area of the substation. The specific simulation parameters are shown in Table 3.

**Table 3:** Simulation parameters.

Serial number	Sports event	Parameters
1	Map width	40 m
2	Minimum safe distance	0.4 m
3	Number of map sampling points	8,000
4	Target point heading angle	45°
5	Map length	45 m
6	Maximum safety distance	0.7 m
7	Initial point heading angle	0°
8	Obstacle impact distance	1.5 m
9	Repulsive gain coefficient	2.0
10	Control interval	0.15 s
11	Gravitational gain coefficient	4.0
12	Electrical Inspection Point Reach Distance Accuracy	0.1 m
13	angle of arrival	11°

In the test, the previously mentioned method of reference^5^ and the method of reference^6^ are used as comparative methods, which are denoted by method 1 and method 2, respectively.

### Experimental procedure and results

**Figure 9:**
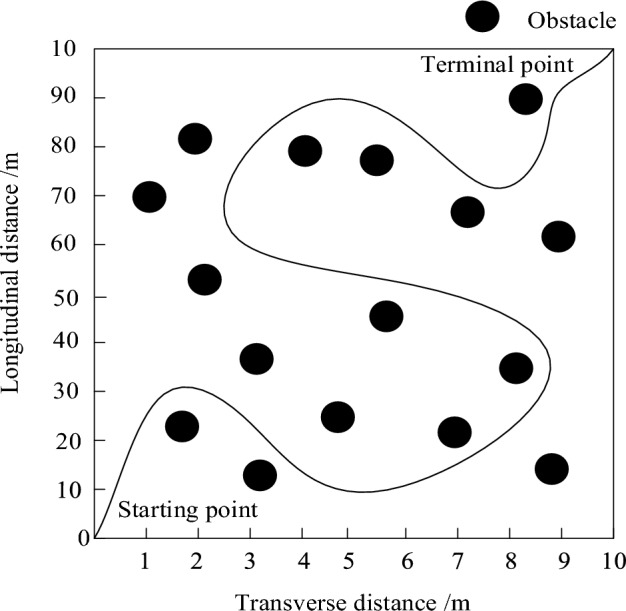
Electrical inspection mobile robot moving preset paths.

Move according to the preset path shown in Fig. 9.

According to the electrical inspection mobile robot moving path in Fig. 8 to complete the robot obstacle avoidance test. The electrical inspection mobile robot movement path can be used to plan the obstacle avoidance path by real-time sensing, recognizing and tracking obstacles, and predicting the dynamic obstacle movement trajectory. The experimental robot is placed at the starting point, and multiple inspection points are set between the starting point and the target point, and three methods are utilized to implement obstacle avoidance navigation control on the experimental robot to ensure that the robot passes through all inspection points. To change the moving speed of the inspection robot, the three methods respectively give control commands, including vertical motion, horizontal motion and rotary motion of the robot base, and compare the inspection paths of the three methods as well as the execution time of the planned paths for obstacle avoidance navigation under different inspection speeds.

**Figure 10:**
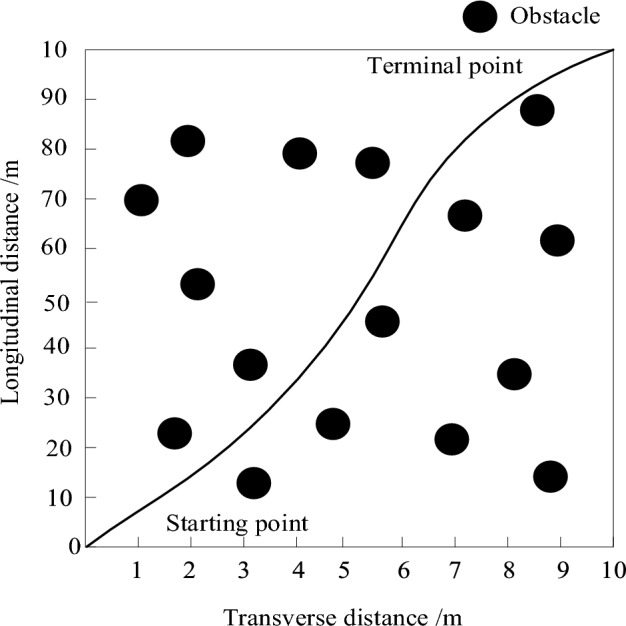
Obstacle avoidance navigation control inspection paths for three methods.

When the patrol speed is 0.15 m/s, the obstacle avoidance navigation control patrol paths of the three methods are shown in Fig. 10.

As can be seen in Fig. 10, the navigation trajectory of this paper's method is smoother and passes through all obstacles, and it is more convenient to move the preset paths than the electrical inspection mobile robot after using the design method, and the electrical inspection mobile robot are able to detect obstacles and plan new paths in time to avoid collision behaviors with obstacles in the process of moving. Under the control of the method in this paper, the electrical inspection mobile robot can successfully avoid obstacles and improve the safety and accuracy of robot operation.

The test results of the execution time of the design method with Method 1 and Method 2 for the planning path of obstacle avoidance navigation at different inspection speeds are shown in Table 4.

**Table 4:** Execution time test results of planning paths for obstacle avoidance navigation.

Patrol inspection speed (m/s)	Design methodology	Method 1	Method 2
0.5	450	500	470
1.0	420	480	450
1.5	230	250	260
2.0	200	230	220
2.5	150	167	157

According to the data in Table 4, it can be seen that under different inspection speeds, there is a difference in the execution time test results of the design method and Method 1 and Method 2 for the obstacle avoidance navigation planning path: the execution time of the design method is slightly lower than that of Method 1 and Method 2 under all inspection speeds, which indicates that the obstacle avoidance navigation control of the design method has a better performance.

**Figure 11:**
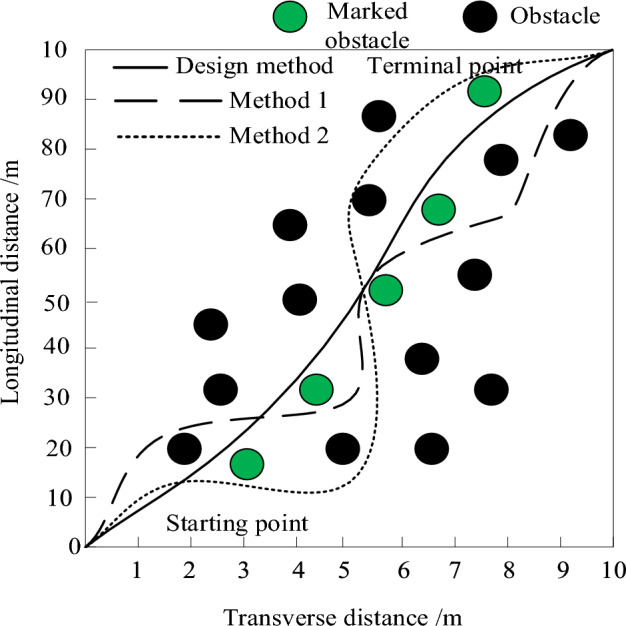
Comparison of the shortest obstacle avoidance path selection under dense obstacles.

In comparative experiments, robots not only need to avoid obstacles completely, but also choose the shortest obstacle avoidance path. Therefore, dense obstacles with interference are placed around the 5 marked obstacles. Among them, the straight-line distance between marked obstacles is the shortest distance, and the three methods are compared for obstacle avoidance path selection under dense obstacles. It is known that all three methods can achieve obstacle avoidance, and the experimental results are shown in Fig. 11.

From Fig. 11, it can be seen that the method proposed in this paper can complete the control of the shortest path along 5 marked obstacle avoidance objects. However, both Method 1 and Method 2 are affected by interfering obstacles, avoiding two marked obstacles and choosing a longer obstacle avoidance path, and Method 2 has a longer path distance than Method 1. Therefore, it can be proven that the method proposed in this article can select the shortest obstacle avoidance path under dense obstacles.

**Figure 12:**
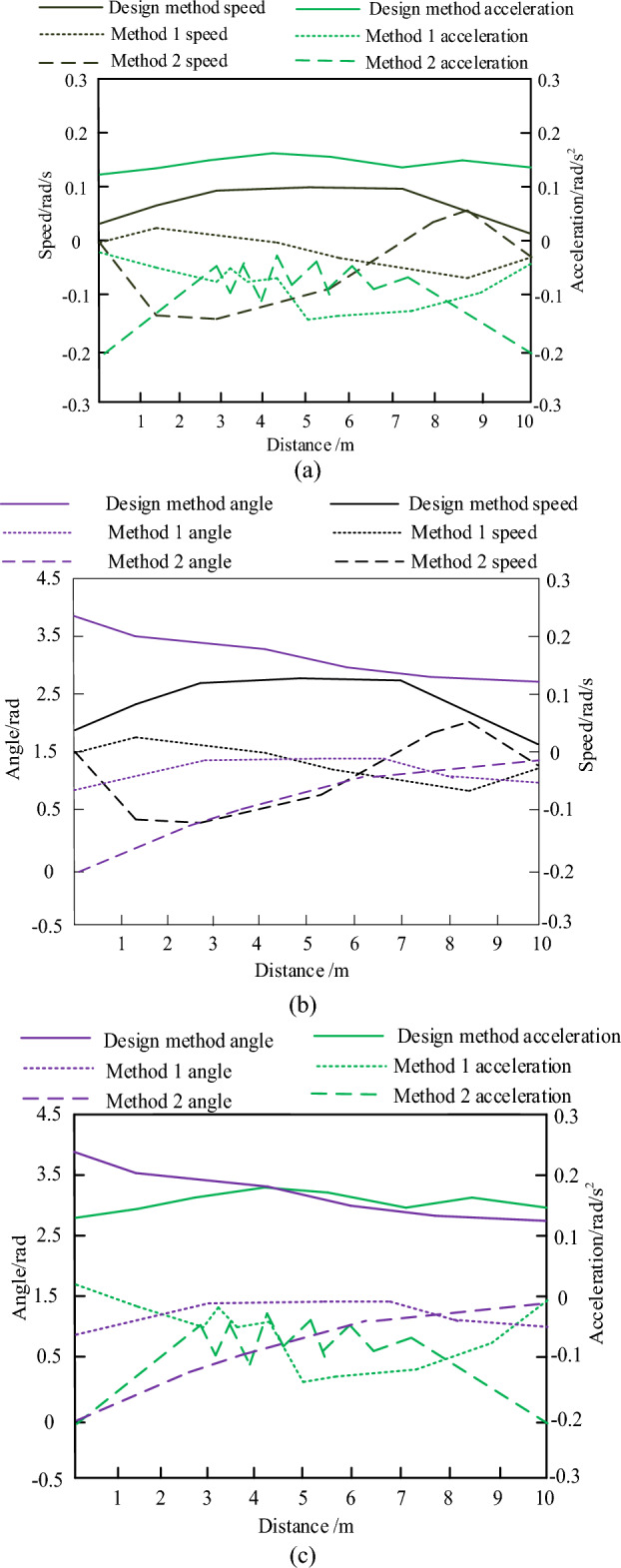
Comparison results under three methods. (**a**) Comparison results of velocity and acceleration under three methods. (**b**) Comparison results of angle and speed under three methods. (**c**) Comparison results of angle and acceleration under three methods.

In order to further verify the effectiveness of the design of the intelligent controller for obstacle avoidance and navigation of the electrical patrol mobile robot based on PLC, continue to design experiments to verify and analyze the method. Methods 1 and 2 are used as comparison methods, and the method in this paper is used as experimental method. Three indicators, namely angle indicator, speed indicator and acceleration indicator, are designed respectively. In the first group of cases, the effects of the three methods are verified with speed indicator and acceleration indicator; In the second group, the effect of the three methods was verified by angle index and speed index; In the third group, verify the effect of the three methods with angle index and acceleration index; The results are shown in Fig. 12.

By comparing the contents of Fig. 12, it can be concluded that the performance of the three methods in terms of velocity and acceleration was evaluated under the first set of experimental conditions. The results show that the designed method exhibits superior results in terms of velocity and acceleration compared with methods 1 and 2, which proves that the designed method has advantages in velocity and acceleration control. In the second set of experimental conditions, the three methods were examined in terms of angle and velocity. The experimental data clearly showed that the design method surpassed Method 1 and Method 2 in terms of angle and speed recognition accuracy, which further verified the accuracy and reliability of the design method in navigation and obstacle avoidance. In the third set of experimental conditions, the validation was conducted using angle and acceleration, and the results showed the superiority of the design method in these two indexes. The design method maintains its superiority even when the evaluation parameters are changed, which shows the robustness and adaptability of the design method.

Based on the above experimental results, it can be concluded that the design method of PLC based intelligent controller for obstacle avoidance and navigation of electrical patrol mobile robot proposed in this paper has obvious advantages in angle, speed and acceleration. These experimental results verify the effectiveness of the designed controller in obstacle avoidance, and show that it can achieve accurate and fast obstacle avoidance navigation in various situations, providing a strong guarantee for the efficient and safe operation of the electrical patrol mobile robot.

## Conclusion

In this study, the design method of intelligent controller for obstacle avoidance and navigation of electric patrol mobile robot based on PLC is deeply discussed and practiced. The precise navigation and obstacle avoidance control of mobile robot are realized by PLC control theory. Through experimental verification, the following conclusions are obtained:

1. The moving path of the electrical inspection mobile robot can be planned by real-time sensing, recognizing and tracking obstacles, predicting the dynamic obstacle movement trajectory, and planning the obstacle avoidance path.
2. The design method shows good adaptability and robustness in complex real-world environments, can successfully avoid obstacles, and improves the safety and accuracy of robot operation.
3. The design method of PLC based intelligent controller for obstacle avoidance navigation of electric patrol mobile robot also improves the efficiency and quality of patrol, and reduces the manual intervention and risk.
4. In the angle, speed and acceleration and other aspects of the method have obvious advantages, in a variety of situations to achieve accurate and rapid obstacle avoidance navigation, for the efficient and safe operation of the electrical inspection mobile robot provides a strong guarantee.

The results of this study are of great significance in promoting the development of electrical inspection automation, which provides strong support for the safe operation of the power system. In the future, we will continue to study the obstacle avoidance and navigation technology of the mobile robot, further optimize the control algorithm, and improve its stability in order to adapt to more complex and changing environments. In addition, we will also expand the application of this method in other fields, such as unmanned vehicles, intelligent transportation and so on. Through continuous research and practice, it is believed that this method will provide more possibilities for the future development of intelligence and automation.

## Data Availability

The datasets used and/or analysed during the current study available from the corresponding author on reasonable request.
